# Low-dose anti-IL 5 treatment in idiopathic hypereosinophilic syndrome: towards a precision medicine approach for remission maintenance

**DOI:** 10.1186/s13023-023-02918-9

**Published:** 2023-09-26

**Authors:** Marco Caminati, Matteo Maule, Roberto Benoni, Claudio Micheletto, Cristina Tecchio, Rachele Vaia, Lucia De Franceschi, Gabriella Guarnieri, Andrea Vianello, Gianenrico Senna

**Affiliations:** 1 Department of Medicine, University of Verona & AOUI Verona, Policlinico GB Rossi, Piazzale L.A. Scuro, 10, 37134 Verona, Italy; 2 Asthma Center and Allergy Unit, AOUI Verona, Policlinico GB Rossi, Verona, Italy; 3 Department of Diagnostics and Public Health, University of Verona, Verona, Italy; 4 Respiratory Unit, AOUI Verona, Policlinico GB Rossi, Verona, Italy; 5 Haematology Unit, AOUI Verona, Policlinico GB Rossi, Verona, Italy; 6 Department of Cardiac Thoracic Vascular Sciences and Public Health, University of Padova, Padua, Italy

**Keywords:** Hypereosinophilic syndrome, Mepolizumab, Remission maintenance

## Abstract

Mepolizumab at the dose of 300 mg/4 weeks has been recently approved as an add-on therapy for patients with uncontrolled hypereosinophilic syndrome (HES) without any identifiable non-hematologic secondary cause. According to the available real-life evidence mepolizumab 300 mg and 100 mg, licensed for severe eosinophilic asthma, are comparable in terms of drug efficacy. However, the clinical rationale for selecting one dose or the other has not been explored. We investigated the efficacy and safety of mepolizumab 100 mg in idiopathic HES (I-HES) patients as a steroid sparing strategy for disease remission maintenance by assessing clinical conditions, blood eosinophil count (BEC) and adverse events at baseline and at 3–6–12 months follow-up. Overall, 11 patients were enrolled (females 4–36%) with a median age of 62 years (IQR 55.0–72.0). At 3-month visit both prednisone daily dose and BEC significantly decreased from baseline, whilst a substantial improvement of Brief fatigue inventory score (BFI) was not recorded before the 6 months assessment. More than 70% of patients completely stopped prednisone at 12-months follow-up, without any flare in terms of BEC and BFI. No adverse event was registered. Although larger studies are needed, our report firstly describes that in a well-defined population, diagnosed with I-HES and in disease remission, low dose mepolizumab is a safe and effective steroid-sparing option for remission maintenance. It suggests that a personalized treatment dose might be explored according to the disease classification and activity at the time of biologic treatment start.

## Introduction

Hypereosinophilic syndrome (HES) is a rare condition defined by persistent blood eosinophil count > 1.5 × 10^9^/L and evidence of eosinophil-related organ damage [1]. Once excluded neoplastic and secondary forms, a heterogeneous group of rare dysimmune conditions can be classified within the mentioned label: myeloid HES (M-HES), associated with molecular abnormalities, including the presence of *FIP1L1::PDGFRA* fusion gene; lymphoid HES (L-HES), in the case flow cytometry reveals the presence of T cell subsets characterized by an aberrant immuno-phenotype (mostly CD3─/CD4 +), with or without clonal T cell receptor (TCR) gene rearrangement; overlap HES, including single-organ eosinophilic disorders (i.e. eosinophilic gastrointestinal disease, eosinophilic pneumonia) and distinct eosinophilic conditions overlapping with HES in their clinical presentation (i.e. EGPA); idiopathic HES (I-HES), not fitting any of the definitions above [1–4]. Regardless of the subtype, HES commonly presents as a very burdensome disease, characterized by non-specific symptoms (fatigue, fever, weight loss, myalgia) and multi-organ impairment mostly involving skin, lungs, digestive tract and heart [2–4]. With the exception of imatinib in selected patients, the traditional pharmacological approach is neither targeted nor specific, relying on oral steroids and cytotoxic therapies [3, 4]. Furthermore common adverse events occurrence and variable efficacy limit their applicability [2].

Mepolizumab, an anti-IL-5 monoclonal antibody, selectively interferes with IL-5 cascade, the most relevant pathway for eosinophils’ generation, development and survival [3]. Initially marketed for severe eosinophilic asthma at the dose of 100 mg every 4 weeks, it has been recently licensed in US and Europe at the dose of 300 mg every 4 weeks as an add on therapy for patients with uncontrolled HES without an identifiable non-hematologic secondary cause [5, 6]. A recently published large international retrospective real-life study portraying the real-word practice in HES management before the approval of mepolizumab 300 mg, pointed out a substantial overlap between mepolizumab 100 and 300 in terms of number of treated patients and drug efficacy [7]. However evidence for supporting the clinical rationale for selecting one dose or the other is currently missing.

We sought to investigate the efficacy and safety of mepolizumab 100 mg in I-HES patients as a steroid-sparing strategy for disease remission maintenance.

## Methodology

We retrospectively reviewed the files of HES patients consecutively referred to our Unit up to October 2022. To be considered for the study, the patients fulfilled criteria for HES according to updated criteria released by the International Cooperative Working Group on Eosinophil Disorders (ICOG-Eo) (absence of an underlying condition causing hyper-eosinophilia, defined as blood eosinophil count > 1500 cells/µl, including a reactive or neoplastic disorder; and: end organ damage attributable to hyper-eosinophilia) [8]. More in detail, all the patients were screened for eosinophilic myeloid neoplasms by *fluorescence in situ* hybridization (FISH) seeking for tyrosine kinase gene fusions, accompanied by NGS-based technique overseeing a panel of 30 genes in order to detect further less frequent mutations and rearrangements including *PDGFRB, FGFR1, JAK 2*. T-cell immuno-phenotyping was also performed in order to exclude aberrant clonal alphabeta T cells commonly (CD3-CD4+) or less commonly (CD3+ CD4+ CD7−, CD3+ CD4− CD8−) related to L-HES.

As further inclusion criterion, at the time of the biologic treatment start stable disease features were requested. In particular, no flares (defined as worsening of HES-related clinical symptoms or a peripheral blood eosinophil count increase requiring an escalation in therapy, unrelated to a decrease in ongoing HES therapy), no major signs of acute disease, and stable prednisone daily intake in the 12 months before enrolment, representing the minimal effective dose for remission maintenance, had to be verified.

The minimal effective dose of prednisone was defined after previous attempts to further tapering the daily intake that resulted in clinical worsening of HES-related symptoms.

Patients receiving other anti-IL 5 drugs or different mepolizumab doses prior to the enrollment were excluded.

Data on clinical conditions, blood eosinophil count and adverse events (AE) recorded at months 0–3–6–12 were considered in order to explore mepolizumab 100 mg/4 weeks efficacy and safety. As an hallmark of clinical response to the treatment, fatigue degree was assessed using a mean of daily Brief Fatigue Inventory (BFI) item 3 values over the 7 days before evaluation (range 0–10 with higher numbers indicating worse fatigue severity) [5, 9]. The steroid-sparing effect of the drug was also evaluated by reviewing the corticosteroid daily dose at the same time-points mentioned above. The study was approved by the local ethic committee.

A descriptive analysis was performed using percentage and median (with interquartile range) for categorical and continuous variable, respectively. Differences in the value of all the three outcome variables (eosinophil count, prednisone dose and BFI) was assessed comparing each time point with baseline via Mann–Whitney-U non-parametric test. A p-value < 0.05 was considered significant. All analyses were performed using the R software (version 4.1.1).

## Results

**Fig. 1:**
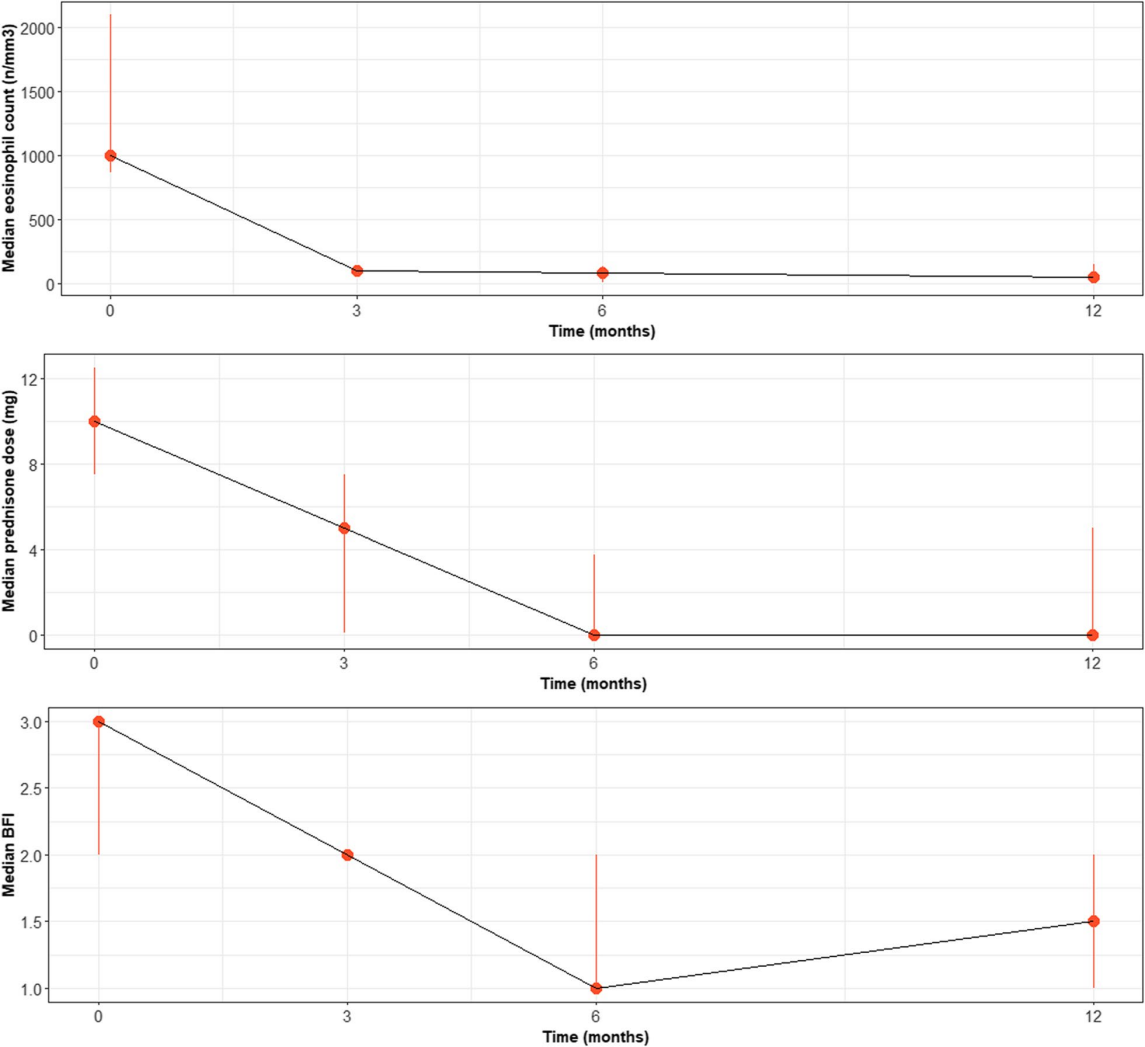
Median value and inter quartile range of the three evaluated outcoms (prednisone dose, eosinophil count and Brief Fatigue Inventory—BFI) at each time point (baseline—t0—and at the 3rd, 6th and 12th month)

Overall, 11 patients were enrolled (females 4–36%) with a median age of 62 years (IQR 55.0–72.0). Clinical characteristics of patients are summarized in Table 1. Constitutional symptoms were almost invariantly present (72% of patients), followed by respiratory manifestations (63% of patients); neurologic, musculoskeletal skin and gastrointestinal signs were less commonly observed. At mepolizumab treatment start visit, the median daily prednisone dose was 10.0 mg (IQR 7.5–12.5), median blood eosinophil level was 1.0 × 10^9^/L (IQR 0.87–2.10) and median BFI value was 3.0 (IQR 2.0–3.0) (Fig. 1).

**Table 1:** Patient characteristics at the time of mepolizumab treatment start

Age—median (IQR)	61.8 (55.0–72.0)
Female—n (%)	4 (36.3)
HES duration (y)—mean ± SD	7.27 ± 6.38
Baseline HES therapy—n (%)	
Prednisone < 10 mg/day	5 (45)
Prednisone ≥ 10 mg/day	6 (55)
Prednisone dose (mg), median (IQR)	10.0 (7.5–12.5)
Baseline blood eosinophil count (cells/μL)—median (IQR)	1000 (870–2100)
HES manifestations—n (%)	
Constitutional (fever, fatigue, weight loss)	8 (72)
Respiratory (dyspnoea, cough, lung infiltrates)	7 (63)
Musculoskeletal (muscle or joint pain)	4 (36)
Neurologic (paraesthesia, hypoesthesia)	5 (45)
Skin (dermatitis, urticaria)	4 (36)
Gastrointestinal (Abdominal pain or bloating, vomiting, diarrhoea)	4 (36)

Under mepolizumab treatment HES was successfully maintained in remission in terms of both symptoms and blood eosinophil count despite the steroid reduction (Fig. 1). Both prednisone daily dose (p = 0.008) and blood eosinophil count (p < 0.001) significantly decreased after 3-month of treatment reaching 5.0 mg (IQR 0.1–7.5) and 0.1 × 10^9^/L (IQR 0.07–0.125), respectively, but not the BFI score (p = 0.086). At 6-month eosinophils count (p < 0.001) and prednisone dose (p < 0.001) were still lower than baseline and BFI significantly decreased from 3 (2–4) to 1 (IQR 1–2) (p = 0.009). Of note one male patient affected by rheumatoid arthritis required an increase of prednisone dose at the 6 months evaluation due to arthritis loss of control following steroid tapering.

Of the 7 patients with the 12 months follow up, 5 (71%) were able to completely withdraw prednisone and all the three outcome variables (eosinophils, prednisone, and BFI) dropped to lower values compared to pre biologic treatment assessment (p < 0.001, p = 0.006, p = 0.024, respectively). No disease flare or AE were registered during the study timeframe and none of the patients needed to withdraw mepolizumab, which was still ongoing at the time of the analysis.

## Discussion

The real word management of HES patients is currently characterized by a heterogeneous approach, even more when considering I-HES [7]. In fact, its diagnostic definition currently relies on the exclusion of other known HES “phenotypes”. In addition its pathobiological background, not yet fully understood, seems to be characterized by multiple dysimmune drivers [1]. It hampers the definition of a major disease target to be pharmacologically addressed and might account for the lack of a treatment option specifically designed for HES. Regarding the last point, the recent approval of mepolizumab 300 mg/4 weeks for HES without an identifiable non-hematologic secondary cause [6] certainly provides a selective strategy supported by a strong clinical rationale, besides the evidence. In fact, targeting eosinophils by robustly interfering with IL-5 cascade addresses the core of the disease pathobiology [1]. However, in the light of a precision medicine approach some practical issue needs to be explored.

Of note, according to the larger real-life study on HES patients’ management published so far, no significant differences in terms of disease control are associated with higher or lower mepolizumab dose [7]. Although the authors did not explore neither the clinical rationale for selecting 100 or 300 mg monthly, nor the characteristics of patients belonging to the different treatment subgroups, a higher pre-biologic treatment steroid intake is reported in patients addressed to mepolizumab 750 mg, which evokes a potential association between the baseline disease severity and the prescribed mepolizumab dose.

However, taken together the authors’ observations suggest that the optimal mepolizumab dosing might be individualized.

Similarly, in EGPA patients a treatment approach with asthma-dose mepolizumab has been explored besides the 300 mg/monthly licensed for that indication [6]. An increasing amount of evidence is supporting the relevance of 100 mg option, especially as a steroid-sparing strategy for maintaining the remission phase [10–12]. Emerging data sustain the off-label successful use of anti IL-5 cascade drugs at asthma dose (i.e. mepolizumab 100 mg/4 weeks and benralizumab 30 mg/8 weeks) in other rare dysimmune eosinophils-driven conditions, including relapsing and/or steroid-dependent idiopathic chronic eosinophilic pneumonia [13].

In our study, mepolizumab 100 mg in patients with I-HES was initiated prior to the approval of 300 mg/monthly for that indication by the regulatory authorities, that represents the main reason for the off-label use of the drug in our population. However, on a clinical ground our treatment choice also relied on the evidence mentioned above, particularly on the data from EGPA patients. In fact, in the light of EGPA as a condition that can be classified under the overlap HES subtype umbrella, that evidence provides a further rationale for exploring a more personalized approach in the management of mepolizumab in HES patients.

Of note, we investigated the low mepolizumab dose as a steroid-sparing agent in a well-defined population, characterized by I-HES diagnosis and disease remission at the time of biologic treatment start. Despite a complete discontinuation or a significant reduction of oral corticosteroids daily dose, blood eosinophil count dropped for every patient at the three-months follow-up and maintained within the normal reference range in the following evaluations. Within the 12 months study time frame, no disease flare was registered and a significant reduction in fatigue severity was observed, despite the significant oral steroids reduction or interruption. In addition, no adverse event was registered during the observation timeframe. Those findings are consistent with the previous studies and trials, although investigating higher mepolizumab doses [5, 14].

Fatigue has been reported as one of the most relevant HES manifestations and its assessment through BFI is part of the clinical outcomes that the published trials have evaluated [5, 15]. In particular, a simplified version including only item 3, which records the worse fatigue level in the last 24 h, has been identified by the authors as the tool for assessing fatigue in the trial study population. Following the same model, and in order to overcome the potential bias related to a single-day detection we evaluated the score values over the 7 days before each follow-up visit. When considering the opportunity to prescribe mepolizumab for HES before it was licensed for that indication, we decided to implement BFI as part of the follow-up assessment of HES patients undergoing mepolizumab 100 mg, taking the opportunity of an already validated Italian version [16]. BFI also provided us a feedback to further sustain the off-label use of the drug, besides its expected impact on blood eosinophil count.

The slight BFI increase we observed between 6- and 12-month follow-up appears to be not significant as the variation occurred within the same interquartile range. However, from a clinical point of view, besides the full adrenal insufficiency, which a gradual corticosteroid tapering usually contributes to prevent, systemic symptoms including weakness and malaise have been described as part of the so-called glucocorticoid withdrawal syndrome [17]. In the light of its impact on BFI items, it might account for the detected BFI variation in our study, occurring in concomitance with oral steroid reduction or withdrawal.

Our findings are not in contrast with the evidence supporting the relevance of mepolizumab 300 mg in HES without an identifiable non-hematologic secondary cause [4–6], but suggest that a personalized treatment dose might be explored according to the disease classification and activity at the time of biologic treatment start.

## Conclusion

Although biased by limitations including the retrospective design, the absence of a control group, the small sample size and the relatively short follow up period, our study suggests that low dose mepolizumab might be considered a safe and effective steroid-sparing option in the remission maintenance of I-HES patients. In addition, our findings support the idea that a tailored targeted-treatment dose could be part of a personalized approach to HES patients. Larger studies are needed to confirm our highlights and to further contribute to structure the approach to a still unclear dysimmune condition.

## Data Availability

The data that support the findings of this study are not openly available due to reasons of sensitivity and are available from the corresponding author upon reasonable request, with permission from the Authors’ Institution and Participants consent. Data are located in controlled access data storage at Verona University Hospital, Allergy Unit and Asthma Center.

## Abbreviations

HES: Hypereosinophilic syndrome
MHES: Myeloid HES
LHES: Lymphoid HES
EGPA: Eosinophilic granulomatosis with polyangiitis
IHES: Idiopathic HES
AE: Adverse events
BFI: Brief Fatigue Inventory
